# Stability Ball Training on Lower Back Strength has Greater Effect in Untrained Female Compared to Male

**DOI:** 10.2478/v10078-012-0052-2

**Published:** 2012-07-04

**Authors:** Chandra Lingesh Sukalinggam, Gabriel Lingesh Sukalinggam, Fajar Kasim, Ashril Yusof

**Affiliations:** 1 Exercise Physiology Laboratory, Sports Centre, University of Malaya.

**Keywords:** stability ball (SB) training, back strength (BS), abdominal strength (AS)

## Abstract

The purpose of this study was to evaluate the effectiveness of short-term stability ball (SB) training on males and females by comparing the strength changes produced in the core muscles. Forty-two previously untrained subjects, mean age = 23.62 ± 2.89 years were matched by their maximum strength (back strength: male = 190–200 kg, female = 45–50 kg and abdominal strength: male = 110–120 kg, female = 35–40 kg 1RM) and randomly placed in either one of these 3 groups; unstable SB group (n = 14), stable floor group (n = 14) and control group (n = 14) who did no exercise. SB training showed greatest improvement (p < 0.001) in back and abdominal strength (25.79 % and 29.51 % respectively), compared with the gain in floor training (FT) back and abdominal strength (10.28 % and 8.47 % respectively). Untrained female subjects achieved a higher percentage of improvement in strength compared to males in both back and abdominal muscles, and this is most evident in the SB training group. It is apparent that performing core training exercises on unstable surfaces stressed the musculature, possibly activating the neuro-adaptive mechanisms that led to the early phase gains in strength.

## Introduction

The core/trunk area could be termed the weak link between the lower and upper extremities of the human body as many people and athletes neglect this area in favour to train other parts of the body. The core refers to the 29 pairs of muscles that support the lumbar-pelvic-hip complex that control movement during force production/transfer and stabilize the lumbar spine, pelvis and kinetic chain in response to balance perturbation during functional movements (Fredericson and Moore, 2005). It is speculated that a strong core allows an individual to fully transfer forces generated from the ground through the lower extremities to the torso, and finally to the upper extremities (Behm et al., 2005; Cissik, 2002).

The lack of core strength and stability can manifest itself by presenting inefficient posture, poor movement technique and also will predispose a person to injury. Since the lower back is often not associated with injury and is placed second only to the common cold as a cause for primary care office visits and direct medical cost which exceeds $25 billion per year (Cypress, 1983; Friedli et al., 1984). Currently most of the available literature on stability ball (SB) relates to functional performance (Anderson and Behm, 2005; Behm et al., 2005; Marshall and Murphy, 2005). However, the studies did not focus on the strength of the lower back.

The training concept proposed in this study represents an important new approach on the effects of short-term SB resistance training in improving core strength, particularly on the lower back. We aimed to investigate the effectiveness of SB training on the core muscles in relation to the enhancement of lower back strength and to determine which gender would receive more benefit from this intervention. We hypothesised that SB training could improve strength of the core muscles and its effectiveness towards females is higher compared to males.

## Material and Methods

### Experimental Approach to the Problem

An experimental design was drawn up to train the specific core muscles for 18 sessions (3 times per week for 6 weeks) using the SB and a padded floor training (FT) as an intervention tool which included exercises such as the Crunch, Supine Leg Lifts, Back Extension, Reverse Back Extension, Supine Rotation, Lateral Crunch, Seated Balance and Core Endurance (Table 1). The control group did no training at all.

### Subjects

Forty-two subjects (n = 42; age = 23.62 ± 2.89 years; body height = 165.89 ± 9.21 cm; body mass = 64.31 ± 14.52 kg) with no prior experience and no history of abdominal/lower back pain or disease volunteered to participate in this study after providing their informed consent (Table 2). The University of Malaya Sports Centre Research Committee approved the research project.

### Procedures

Ninety eight (n = 98) subjects were screened during pre-test of a 1RM back extension test (Vision ST220 Abs/ Back Machine) and a 1RM abdominal curl test (Vision ST220 Abs/ Back Machine) 48 hours prior to commencement of the training to meet the criterion selected (Back strength: male = 190–200 kg, female = 45–50 kg and Abs Strength: male = 110–120 kg, female = 35–40 kg 1RM). The selected subjects (n = 42; 21 males and females) were then randomly assigned to 3 groups (control group = 14, SB group = 14 and padded FT group = 14). A three day diet monitoring procedure was employed to control dietary influence on results obtained. They were required to maintain their common daily activity lifestyle and food intake. The 1RM tests were repeated after the completion of the 6 weeks intervention program to determine the back and abdominal strength. Participants performed 3 trials and the best result was recorded. The RPE was administered immediately after every trial with a reading from 0 to 10 (easy to hard). In order to avoid muscular fatigue, a 5-minute rest period was allowed between trials. Participants were verbally encouraged during maximum efforts. All data for each subject were collected during a single session.

All core training techniques and cadences were taught to each subject and sufficient practice was allowed for the rhythm of the movement to be properly learned. Each participant was given time to familiarise with the exercise protocol before the commencement of the intervention program. All exercises cadence were kept in time with a metronome (1 repetition per s) with a 30 s recovery period between sets, except for Seated Balance and Core Endurance exercises which require a 60 s rest period (Table 1). Each session lasted approximately 30 to 40 minutes and started with a warm up protocol that included spinal mobility exercises and stretches on the SB or floor, such as the cat/camel, a back arching exercise done while on the hands and knees to increase mobility and blood flow to the spinal region.

### Statistical Analyses

The effects of different treatment factors (SB, FT and control) versus gender were determined by using 2-Way Repeated Measure Analyses of Variance (ANOVA). The data has met all the assumptions for linear statistic and the Shapiro-Wilk test of homogeneity was carried out to assess variance between groups. A Bonferroni post-hoc test was carried out to determine the significance of pair wise comparison. Statistical significance was set at p < 0.05 for hypothesis testing. All values were reported as mean ± SD.

## Results

Comparisons of delta or relative changes between pre and post-test for back strength and abdominal strength of the 3 groups are presented in Table 3.

**Figure 1:**
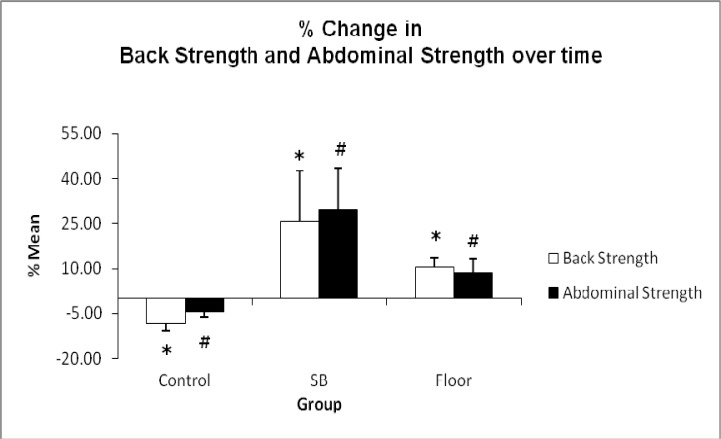
Comparison between pre-test and post-test mean values in all group dependent variable between control and experimental groups (SB and floor) before and after 6 weeks of intervention (* p < 0.05 for Back Strength and # p ≤ 0.05 for Abdominal Strength)

**Figure 2:**
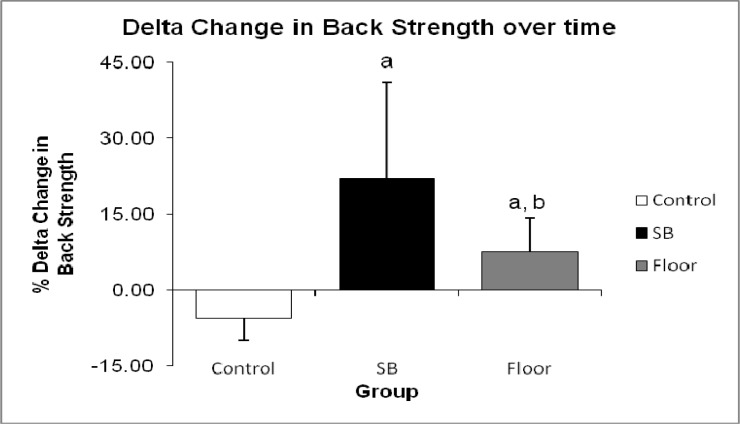
Comparison between groups of delta change in values pre-test and post-test for back strength (a denotes p < 0.05 between Control Group and SB Group, also between Control Group and Floor Group, and b denotes p < 0.05 between SB Group and Floor Group)

**Figure 3:**
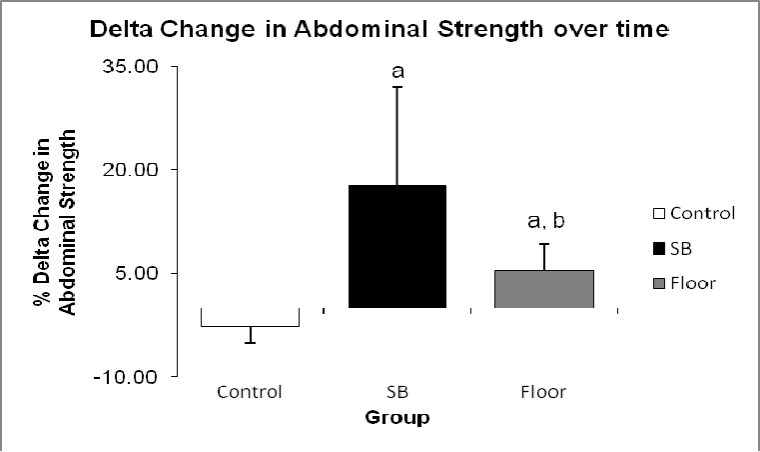
Comparison between groups of delta change in values pre-test and post-test for abdominal strength (a denotes p < 0.05 between Control Group and SB Group, also between Control Group and Floor Group, and b denotes p < 0.05 between SB Group and Floor Group)

Overall comparison of female and male % changes in back strength and abdominal strength between pre and post-test in all the groups were statistically significant (p < 0.05) as shown in Figure 1. SB training showed greatest improvement in back strength (25.79 %) and abdominal strength (29.51 %), compared to the gain in FT back strength (10.28 %) and abdominal strength (8.47 %) with p < 0.001. Figure 2 shows the relative changes (post minus pre-test) in back strength which further elucidate the greater improvement in SB group (27.64 ± 19.99 kg) compared to FT (9.00 ± 7.36 kg) at p < 0.001. In abdominal strength of the SB groups showed significant improvement (21.79 ± 14.92 kg) compared to FT (5.86 ± 4.49 kg) as shown in Figure 3.

**Figure 4:**
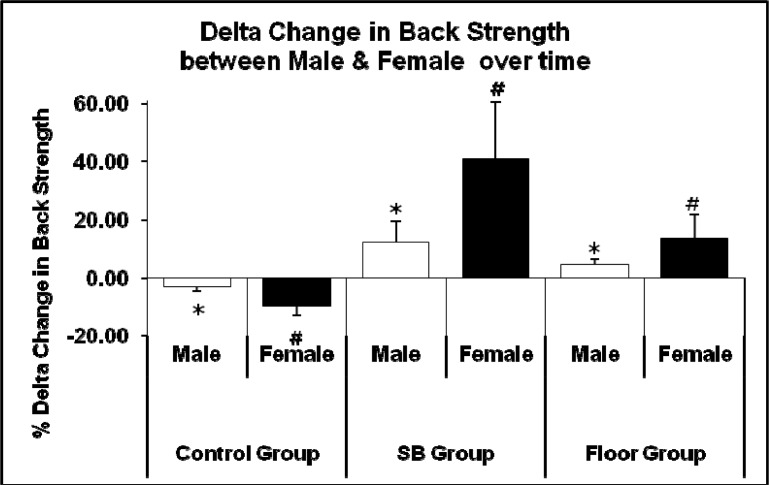
Comparison of mean delta change in values pre-test and post-test between male and female gender in back strength (* p < 0.05 for male, # p < 0.05 for female)

**Figure 5:**
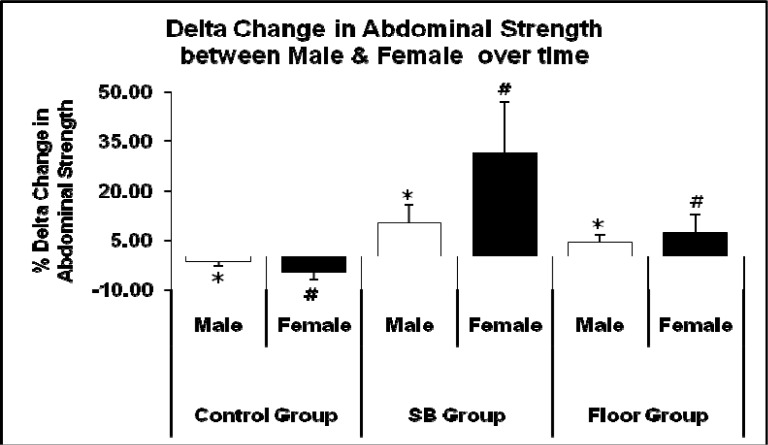
Comparison of mean delta change in values pre-test and post-test between male and female gender in abdominal strength (* p < 0.05 for male, # p < 0.05 for female)

Females showed greater improvements in back and abdominal strength than males when gender comparison was made. Females in the SB group showed higher improvement (40.71 %) compared to males (12.00 %) in back strength as shown in Figure 4. In abdominal strength, females also showed greater improvement (31.57 %) compared to males (10.31 %) as shown in Figure 5.

## Discussion

The 6-week core intervention program in this study resulted in significant increases in back and abdominal strength between the SB group (unstable) and the FT group (stable) as seen in Figure 1. To our knowledge, only one study has shown comparable results following a 6-week SB training specifically designed for core activation where significant increases in back and abdominal strength were observed (Stanton et al., 2004).

The SB group (female and male) showed a two-fold increase in back strength while abdominal strength increased three folds compared to the floor group (Figure 1). The positive changes may be attributed to the implementation of the SB core training which improves core stability, and portrays the complex interaction of passive (joint articulations and spinal ligaments) and active (neural and muscular) subsystems that maintain intervertebral neutral zones within the physiological limits (Panjabi, 1992). Research also suggests the adaptation gained from SB training is likely to result in better coordination of synergistic and stabilizer core muscles (Rutherford and Jones, 1986). The muscles that make up the core can be divided into *local* and *global* groups based on location and attachment sites (Bergmark, 1989).

The benefits of performing resistance exercises on unstable equipment originated from research on muscle activation and methods of preventing or rehabilitating low back, knee, and ankle injuries (Fitzgerald, 2000; Nadler et al., 2002; McGill et al., 2003; Verhagen et al., 2004). Even though the movement patterns on the SB and FT group may look similar, the underlying neural adaptations such as the increase in nervous system activation, more efficient neuromuscular recruitment patterns, improved synchronization of motor units, lowering of neural inhibitory reflexes and proprioceptive feedback may be completely different (Panjabi, 1992; Staron et al., 1994). This short-term exposure to SB training resulted in significant improvement in the sway control, core stability and inhibition of inappropriate motor responses by altering sensory input (Wolfson et al., 1993). Other studies have indicated that as the degree of instability increased, the degree of core muscle activity will increase proportionally (Anderson and Behm, 2005; Behm et al., 2005; Marshall and Murphy, 2005).

Statistically significant differences were observed in the pre and post-test results of back strength and abdominal strength within and between groups in this study (Figures 2 and 3). The SB group showed a three-fold increase in back strength and abdominal strength over the course of the intervention period as compared to the FT group. One factor affecting back strength is muscle force-stiffness which may influence joint stability of the spine. The spine must achieve sufficient stability to handle any imposed loads without risk of buckling (Cholewicki and McGill, 1996; Cholewicki et al., 2000; 2002) and that stability is achieved only with balancing of stiff muscles around the spine (Brown and McGill, 2005). Most core muscles create moments about the 3 orthopedic axes of the spine (McGill, 1991). Therefore, the muscle turns on significantly when the motion of the spine is created before other muscles to create continuity of force/moment through the stable core segment linkage thus reducing energy leaks and increasing force production (McGill et al., 2009). Initial scientific support for the SB training was noted in relation to specific phases of lumbar rehabilitation when both the rectus abdominus and the external oblique muscles were seen to be activated during abdominal crunch exercises (Vera-Garcia et al., 2000). Another research indicates that the lumbar multifidus and transverse abdominus may be involved in controlling spinal stability (Cresswell et al., 1994).

Interactions could also be seen between gender and back strength (Figure 4) and abdominal strength (Figure 5) in the SB and FT group. Overall, females showed a higher improvement in back and abdominal strength in both the SB and FT group as compared to their male counterparts (comparison between intervention groups also shows that the SB group had a higher increase in back and abdominal strength as compared to those in the FT group). Similar cases were seen in other studies, where untrained females showed higher improvements compared to males (Brown and Wilmore, 1974; Mayhew and Gross, 1974; Hunter, 1985). This may be due to the fact that women tend to have more slow-twitch muscle fibres than men, which may influence more stiffening of the local muscle groups of the core thus producing more significant force transfer. Previous research indicated that women have a greater ratio of slow-twitch to fast-twitch fiber area compared to their male counterparts (Bell and Jacob, 1990; Shephard, 2000; Staron et al., 1994). We are also not ruling out the fact that their initial values were lower than in males.

It is evident that performing core training exercises on unstable surfaces stressed the musculature and possibly activated the neuro-adaptive mechanisms that led to the early phase gains in stability and proprioceptive activity (Behm et al., 2002). The research supports the hypothesis the SB training improves strength of the core muscles more significantly than FT and these changes are more evident in female subjects.

## Practical Application

For coaches or personal trainers, the use of SB either alone or as an adjunct to other physical exercise modalities such as dumbbells and barbells, cable devices and resistance bands will enhance core strength and stability. The difficulty of these exercises can be modified by adjusting base of support or load distribution. The individual may less likely be injured if there is a more efficient control of the upper and lower body muscles by having a stronger core musculature. The core musculature training progression should work from the inside-out and focus on optimizing the function of the local system before emphasizing movements that utilize the global system. SB could also be used as a training tool for rehabilitation after injury which may increase recovery rate and regain strength.

## Figures and Tables

**Table 1:** Core training program 3 times per week for 6 weeks

CORE TRAINING PROGRAM				

Set × Reps × Recovery (sec)				
No.	Description	Weeks 1 and 2	Weeks 3 and 4	Weeks 5 and 6
1	Crunch	3 × 20 × 30	2 × 30 × 30	1 × 60 × 30
2	Supine Leg Lifts	3 × 20 × 30	2 × 30 × 30	1 × 60 × 30
3	Back Extension	3 × 20 × 30	2 × 30 × 30	1 × 60 × 30
4	Reverse Back Extension	3 × 20 × 30	2 × 30 × 30	1 × 60 × 30
5	Supine Rotation	3 × 20 × 30	2 × 30 × 30	1 × 60 × 30
6	Side Bend	3 × 20 × 30	2 × 30 × 30	1 × 60 × 30
7	Seated Balance	1 × 60 sec × 30	1 × 90 sec × 30	1 × 120 sec × 30
8	Core Endurance	1 × 60 sec × 30	1 × 90 sec × 30	1 × 120 sec × 30

**Table 2:** Descriptive statistics for all 3 groups participating in a 6-week training program (n = 42)

Group Dependent Variables	n = 14	n = 14	n = 14

	Group A (Control)	Group B (SB)	Group C (FT)

	Mean ± SD	Mean ± SD	Mean ± SD
Age (years)	22.50 ± 0.52	24.07 ± 3.41	24.29 ± 3.52
Height (cm)	165.55 ± 8.59	165.57 ± 10.47	166.54 ± 9.15
Weight (kg)	62.51 ± 13.69	66.37 ± 15.91	64.04 ± 14.69

**Table 3:** Comparison of mean delta change of pre- and post-test values for all groups

Group Dependent Variables	n = 14	n = 14	n = 14	p

	Group A (Control)	Group B (SB)	Group C (Floor)	
Back Strength	−6.71 ± 3.71^*^	27.64 ± 19.99^*^	9.00 ± 7.36^*^	0.001
Abdominal Strength	−2.93 ± 1.77^*^	21.79 ± 14.92^*^	5.86 ± 4.49^*^	0.001

* significant where p < 0.05

## References

[b1-jhk-33-133] Anderson K, Behm D (2005). The impact of instability resistance training on balance and stability. J Sports Med.

[b2-jhk-33-133] Anderson K, Behm D (2005). Trunk muscle activity increases with unstable squat movements. Can. J Appl Physiol.

[b3-jhk-33-133] Behm DG, Leonard AM, Young WB, Bonsey WAC, Mackinnon SN (2005). Trunk muscle electromyographic activity with unstable and unilateral exercises. J Strength Cond Res.

[b4-jhk-33-133] Behm DG, Kenneth A, Curnew RS (2002). Muscle force and activation under stable and unstable conditions. J Strength Cond Res.

[b5-jhk-33-133] Bell DG, Jacobs I (1990). Muscle fibre area, fibre type and capillarization in male and female body builders. Can J Sports Sci.

[b6-jhk-33-133] Bergmark A (1989). Stability of the lumbar spine: A study in mechanical engineering. Acta Orthop Scand.

[b7-jhk-33-133] Brown C, Wilmore J (1974). The effects of maximal resistance training on the strength and body composition of women athletes. Med Sci Sports.

[b8-jhk-33-133] Brown SH, McGill SM (2005). Muscle force-stiffness characteristics influence joint stability. Clin Biomech.

[b9-jhk-33-133] Cholewicki J, McGill SM (1996). Mechanical stability of the in vivo lumbar spine: implications for injury and chronic low back pain. Clin Biomech.

[b10-jhk-33-133] Cholewicki J, Simons A, Radebold A (2000). Effects of external trunk loads on lumbar spine stability. J. Biomech.

[b11-jhk-33-133] Cholewicki J, Vanvliet JJ (2002). Relative contribution of trunk muscles to the stability of the lumbar spine during isometric exertions. Clin. Biomech.

[b12-jhk-33-133] Cissik JM (2002). Programming abdominal training, part one. Strength Cond J.

[b13-jhk-33-133] Cresswell AG, Oddsson L, Thorstenson A (1994). The influence of sudden perturbations on trunk muscle activity and intra abdominal pressure while standing. Exp Brain Res.

[b14-jhk-33-133] Cypress BK (1983). Characteristics of physician visits for back symptoms: a national perspective. Am J Public Health.

[b15-jhk-33-133] Fredericson M, Moore T (2005). Core stabilization training for middle and long-distance runners. New Stud Athletics.

[b16-jhk-33-133] Fredericson M, Moore T (2005). Muscular balance, core stability, and injury prevention for middle- and long-distance runners. Phys Med Rehab Clin North Am.

[b17-jhk-33-133] Friedli WG, Hallet M, Simon SR (1984). Postural adjustments associated with rapid voluntary arm movements, I: electromyographic data. J Neurol Neurosurg Psychiatry.

[b18-jhk-33-133] Hunter GR (1985). Changes in body composition, body build and performance associated with different weight training frequencies in males and females. Nat Strength Cond Assoc J.

[b19-jhk-33-133] Marshall PW, Murphy BA (2005). Core stability exercises on and off a Swiss ball. Arch Phys Med Rehabil.

[b20-jhk-33-133] Mayhew JL, Gross PM (1974). Body composition changes in young women with high intensity weight training. Res Q.

[b21-jhk-33-133] McGill SM, Karpowicz A, Fenwick CMJ (2009). Ballistic abdominal exercises: muscle activation patterns during three activities along the stability/mobility continuum. J Strength Cond Res.

[b22-jhk-33-133] McGill SM, Grenier S, Kavcic N, Cholewicki J (2003). Coordination of muscle activity to assure stability of the lumbar spine. J Electromyogr Kinesiol.

[b23-jhk-33-133] McGill SM (1991). The kinetic potential of the lumbar trunk musculature about three orthogonal orthopaedic axes in extreme postures. Spine.

[b24-jhk-33-133] Nadler SF, Malanga GA, Bartoli LA, Feinberg JH, Prybicien M, Deprince M (2002). Hip muscle imbalance and low back pain in athletes: influence of core strengthening. Med Sci Sports Exerc.

[b25-jhk-33-133] Panjabi MM (1992). The stabilizing system of the spine. Part I. Function, dysfunction, adaptation, and enhancement. J Spinal Disord.

[b26-jhk-33-133] Panjabi MM (1992). The stabilizing system of the spine. Part II. Neutral zone and instability hypothesis. J Spinal Disord.

[b27-jhk-33-133] Rutherford OM, Jones DA (1986). (1986) The role of learning and coordination in strength training. Eur J Appl Physiol.

[b28-jhk-33-133] Shephard RJ (2000). Exercise and training in women. Part I: Influence of gender on exercise and training responses. Can J Appl Physiol.

[b29-jhk-33-133] Stanton R, Reaburn PR, Humphries B (2004). The effect of short-term Swiss ball training on core stability and running economy. J Strength Cond Res.

[b30-jhk-33-133] Staron RS, Karapondo DL, Kraemer WJ, Fry AC, Gordon SE, Falkel JE, Hagerman FC, Hikida RS (1994). Skeletal muscle adaptations during early phase of heavy resistance training in men and women. J Appl Physiol.

[b31-jhk-33-133] Vera-Garcia FJ, Grenier SG, McGill SM (2000). Abdominal muscle response during curl-ups on both stable and labile surfaces. Phys Ther.

[b32-jhk-33-133] Verhagen E, Van Der Beek A, Twisk J, Bouter L, Bahr R, Mechelen WV (2004). The effect of a proprioceptive balance board training program for the prevention of ankle sprains. Am J Sports Med.

